# Concurrent reactive arthritis and myelitis – a case report

**DOI:** 10.1186/1755-7682-2-17

**Published:** 2009-06-02

**Authors:** Mukul P Agarwal, Subhash Giri, Vishal Sharma, Gaurav Bhardwaj

**Affiliations:** 1 Department of Medicine, University College of Medical Sciences, Delhi, India

## Abstract

Reactive arthritis is a post infectious multisystem illness which usually occurs after episodes of diarrhoea or urinary tract infections. It can cause many manifestations other than the musculoskeletal system including skin, urogenital system and eyes. However the central nervous system is only occasionally involved. We discuss the case of a 32 year old male who presented with myelitis in association with reactive arthritis.

## Background

Reactive arthritis is a post infectious form of joint involvement which usually antedates episodes of diarrhoea or urinary tract infections. It can cause many manifestations involving the musculoskeletal system, skin, urogenital system, eyes or heart. Central nervous system manifestations are infrequent. Here we present the case of a middle aged male who had spinal cord involvement in form of myelitis in association with reactive arthritis. We could come across only two past reports of such coexistence.

## Case presentation

A 32 year old male presented to us with complaints of pain and swelling of multiple joints of one month duration. The symptoms started in the right knee but soon involved the left knee, both ankles and hips. The pain was associated with morning stiffness which persisted for around one hour. Small joints of the hand were not involved. There were no ocular complaints. The patient also complained of backache which began at around the same time as the joint pain. No complaints of numbness, parasthesias, root pain, falls, and bladder or bowel symptoms were present. A fortnight later the patient developed skin lesions which began as fluid filled lesions over the back and later involved all limbs and the forehead. The patient also complained of some lesions over the penis. The patient gave history of multiple unprotected extramarital sexual contacts. He gave a history of dysuria prior to the onset of joint pains. He was a chronic smoker and occasionally took alcohol.

**Figure 1:**
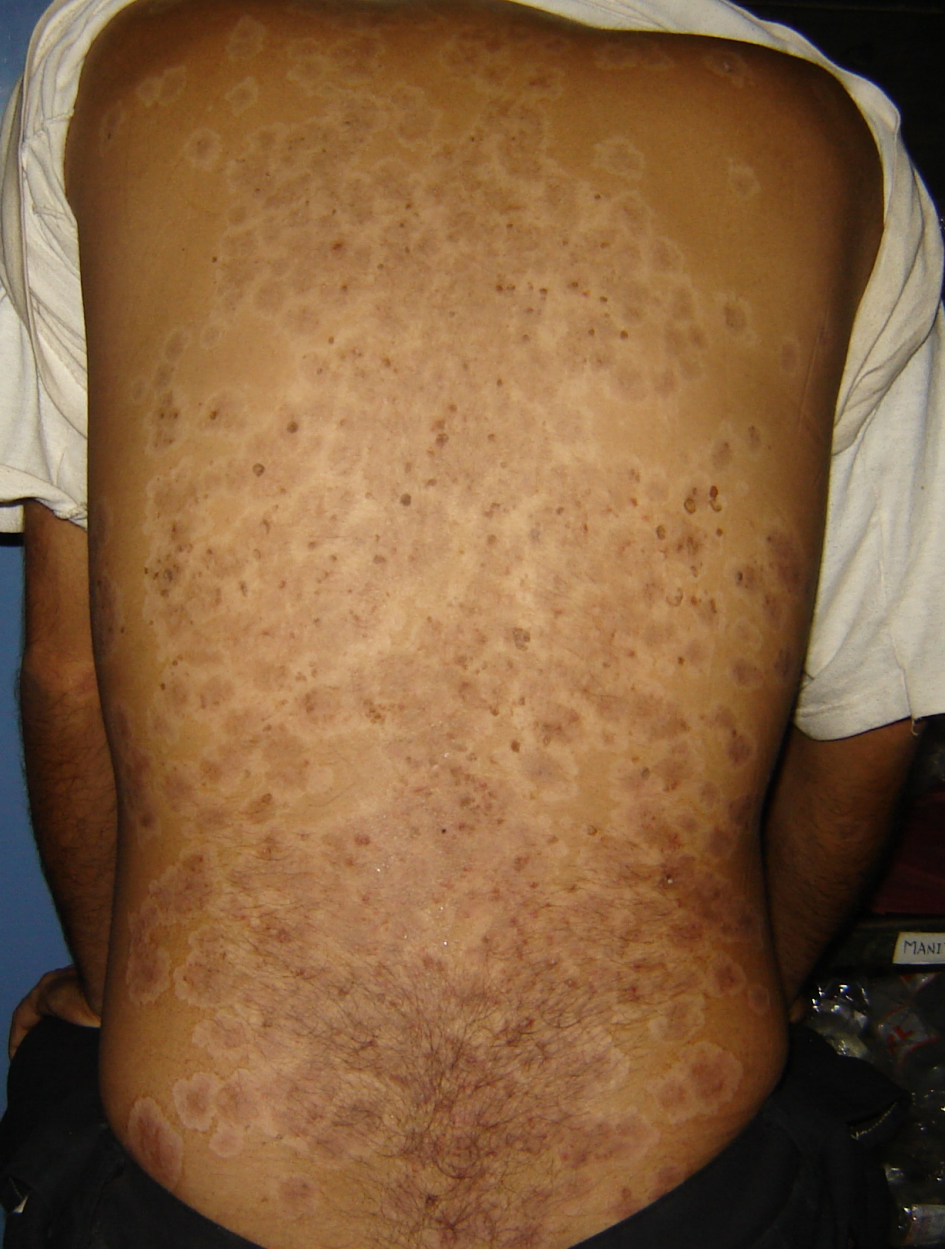
**The rash at patient's back**.

On examination he appeared pale but had no lymphadenopathy. He had multiple discrete to coalescent erythematous plaques with brown crust and dirty white scales on the back, forehead, all four limbs and buttocks (Figure 1). Grattage test was positive but Auspitz sign was negative. Toe nails had nail plate discoloration and subungual hyperkeratosis. Multiple pin head sized erosions were present over glans penis and corona of penis in a circumferential manner suggestive of circinate balanitis. The chest expansion was reduced. The examination of cardiovascular and gastrointestinal system was normal. Testing for power was limited due to pain in joints. However, the patient had lower limb hypereflexia, sustained ankle clonus and extensor plantars. There was no evidence of any sensory loss, root pains, bladder or bowel involvement. Examination of spine revealed tenderness at second to sixth thoracic and the lumbar vertebrae. Straight leg raising test was negative. Modified Schober test revealed lumbar expansion of 3 cm. Locomoter system examination revealed swollen knees with a positive patellar tap. Direct pressure over the sacroiliac joints elicited tenderness. Joint movements were restricted at both ankles and knees.

**Figure 2:**
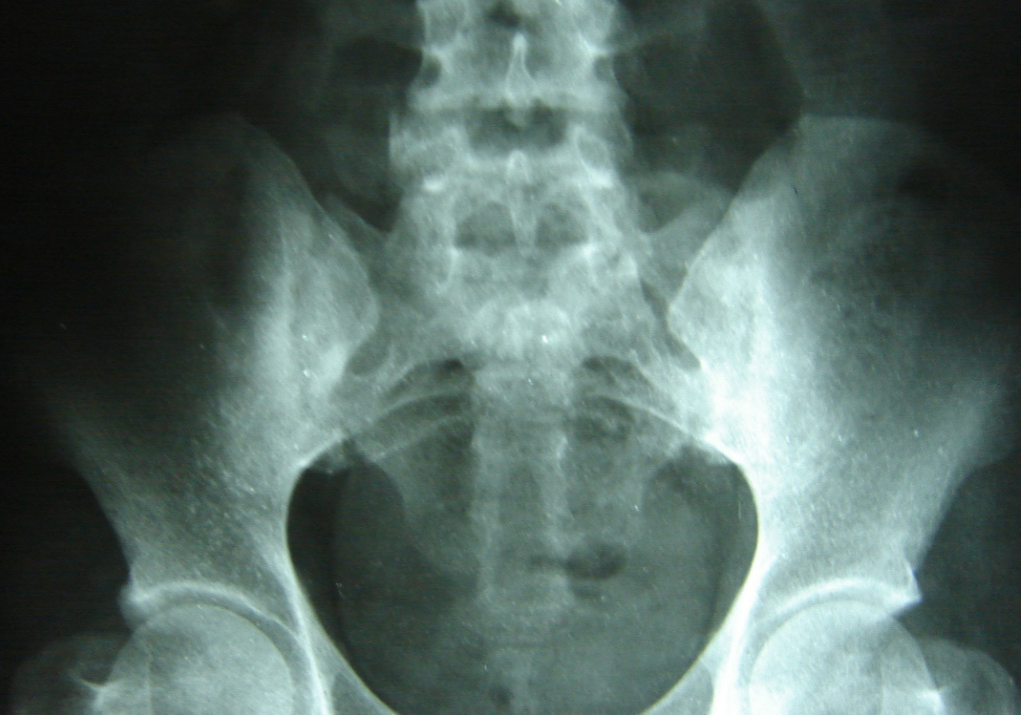
**X-Ray of pelvis showing sacroilitis**.

**Figure 3:**
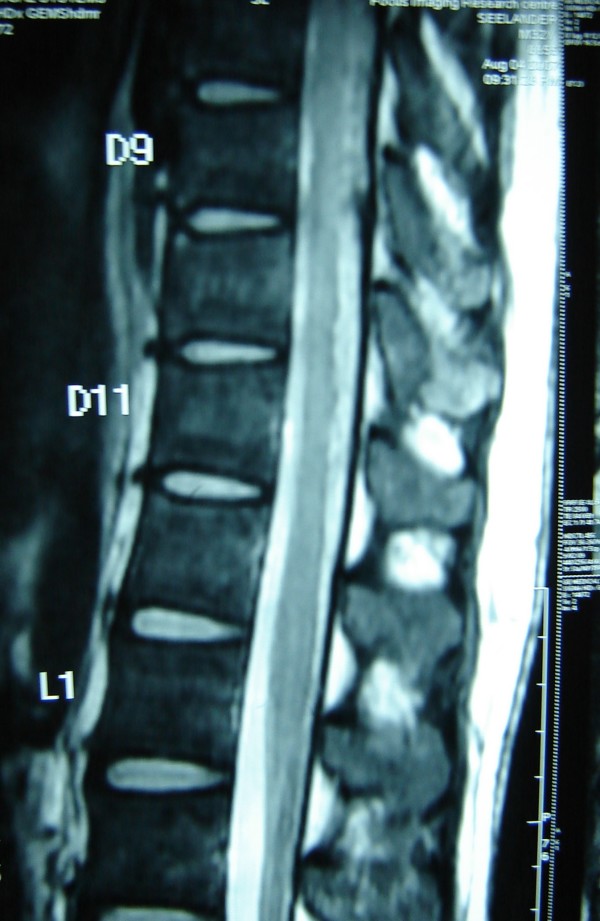
**MRI of spine showing myelitis**.

He was anemic (Hemoglobin-8.7 g/dl) with a microcytic hypochromic picture. He had normal leukocyte count, raised Erythrocyte Sedimentation Rate (60 mm in 1st hour), normal serum uric acid, liver and renal functions. Rheumatoid factor was negative and ASLO titer was not raised. Semi quantitative assay for CRP titer was positive (>6 mg/L). Serology for HIV, HBV, HCV, syphilis and HSV-1 and 2 was negative. ANA was negative. HLA-B 27 was positive. Urethral smear revealed multiple neutrophils but urine culture revealed no growth. X rays of chest, hands, knees and feet were normal. However roentgenograms of spine and sacroiliac joints revealed sclerosis at both sacroiliac joints and marginal syndesmophytes on lumbar spine (Figure 2). Echocardiography revealed mild aortic root dilatation and aortic regurgitation. USG abdomen was suggestive of cystitis. Skin biopsy was consistent with psoriasiform dermatitis. MRI spine revealed multiple hyperintensities in the spinal cord in the region of T9–T11 (Figure 3) and in the medullary conus. The patient could not afford Chlamydial PCR. Lumbar puncture was done and the cerebrospinal fluid was clear with 35 cells/μL predominantly lymphocytes, with protein of 48 mg/dL and glucose 52 mg/dL. No oligoclonal band was seen on electrophoresis. Gram stain and cultures of CSF were negative.

## Conclusion

In a young male presenting with acute inflammatory additive polyarthritis with evidence of spondyloarthropathy developing after a urinary tract infection, the possibility of acute Reactive Arthritis has to be considered. The presence of psoriasiform rash and circinate balanitis raised the possibility of psoriatic arthritis. However, the appearance of joint symptoms prior to the rash is only occasionally seen in psoriasis. Also, the HLA – B 27 positivity favors Reactive Arthritis. HLA – B 27 can be positive in axial involvement in psoriatic arthritis but is less common with appendicular involvement. The presence of enthesitis (tenderness over spine), aortic root dilatation, sacroilitis, circinate balanitis and skin lesions beginning as vesicles were all consistent with Reactive Arthritis[1].

However the interesting finding in this case was the presence of bilateral lower limb hypereflexia, extensor plantar response and presence of clonus raising the possibility of compressive myelopathy due to spondylitis. The MRI of the dorsal spine revealed evidence of patchy myelitis. Interestingly the occurrence of both the reactive arthritis and myelitis was temporally related. There are only a few previous reports of coexistence of Reactive Arthritis with myelitis. In one (Neuromyelitis optica) this was explained as probably coincidental [2]. Here a lady who had been having recurrent episodes of myelitis later developed reactive arthritis. Another report has indicted Reiter's as a cause of myelopathy in a 21 year old male [3]. In this case a young male developed myelopathy associated with reactive arthritis secondary to a urinary tract infection.

The pathogenesis of both Reactive Arthritis and myelitis is explained on the basis of autoimmune phenomenon occurring in response to some infection. Multiple organisms are implicated in causation. Reactive arthritis is known to follow genitourinary and gastrointestinal infections, usually bacterial. Postinfectious myelitis usually follows viral infections. However myelitis can follow bacterial infections including Campylobacter and Yersinia [4]. It is likely that a single preceding insult was responsible for the causation of Reactive Arthritis and myelitis in this case. Unfortunately no etiogic agent could be identified. The patient responded to high dose broad spectrum antibiotics, intravenous methylprednisolone followed by oral prednisolone. The hypereflexia disappeared and the plantars became flexor.

This case highlights the concurrent occurrence of Reactive Arthritis and myelitis. To dismiss this merely as a coincidence might not be appropriate in view of the common mechanisms involved in causation of these two phenomena. Preceding infection and autoimmune response to the infection are believed to be responsible for both and it is attractive to indict a single agent in causation

## Consent

Written informed consent was obtained from the patient for publication of this case report and any accompanying images. A copy of the written consent is available for review by the Editor-in-Chief of this journal.

## Competing interests

The authors declare that they have no competing interests.

## Authors' contributions

MPA and SG were involved in conception and design, revising the manuscript and have given final approval. VS and GB were involved in acquisition of data, drafting the manuscript and have given final approval of the version to be published.
